# Efficacy, Stability, and Biosafety of Sifuvirtide Gel as a Microbicide Candidate against HIV-1

**DOI:** 10.1371/journal.pone.0037381

**Published:** 2012-05-16

**Authors:** Liangzhu Li, Yinyin Ben, Songhua Yuan, Shibo Jiang, Jianqing Xu, Xiaoyan Zhang

**Affiliations:** MOH/MOE Key Laboratory of Medical Molecular Virology and Institute of Medical Microbiology, Shanghai Public Health Clinical Center and Institutes of Biomedical Sciences, Shanghai Medical College, Fudan University, Shanghai, China; Scripps Research Institute, United States of America

## Abstract

Sifuvirtide is a proven effective HIV-1 entry inhibitor and its safety profile has been established for systemic administration. The present study evaluated the potential of sifuvirtide formulated in a universal gel for topical use as a microbicide candidate for preventing sexual transmission of HIV. Our data showed that sifuvirtide formulated in HEC gel is effective against HIV-1 B, C subtypes, CRF07_BC and CRF01_AE, the latter two recombinants represents the most prevalent strains in China. In addition, we demonstrated that sifuvirtide in gel is stable for at least 8 weeks even at 40°C, and did not cause the disruption of integrity of mucosal epithelial surface, or the up-regulation of inflammatory cytokines both *in vitro* or *in vivo*. These results suggest that sifuvirtide gel is an effective, safe and stable product, and should be further tested as a vaginal or rectal microbicide in pre-clinical model or clinical trial for preventing HIV sexual transmission.

## Introduction

Recent report from Joint United Nations Programme on HIV/AIDS (UNAIDS) showed that the global HIV-1 new infections have declined by 19% in year 2010 compared with that in year 2009 [1]. Since the majority of new infections occur through sexual intercourse, the use of microbicides applied to vaginal or rectal mucosa is an important approach to prevent the sexual transmission. Recently, the focus in microbicide development is shifting from broad spectrum microbicide products with nonspecific action mechanisms, such as surfactants or polyanions, to those that can specifically inhibit HIV-1 infection, including reverse transcriptase inhibitors (RTIs) [2], [3]. A recent clinical trial showed that 1% tenofovir (TFV), a gel version of the nucleotide reverse transcriptase inhibitor (NRTI), provided moderate protection against sexually transmitted HIV-1 [4], which for the first time demonstrated that microbicide is a promising approach to restrain HIV spreading.

HIV-1 infection could be dissected into multiple consecutive steps, including binding, fusion, entry, reverse-transcription, integration, transcription, translation and cleavage, assemble and budding; Candidate drugs targeting procedures other than reverse transcription are also likely to be effective if used as active components in microbicide candidate. Similar to highly active antiretroviral therapy (HAART), the most effective strategy for microbicide development may need to combine several antiretroviral drugs targeting at different steps during HIV-1 life cycle. Since HIV-1 viral entry is considered as an important target in combating HIV/AIDS infections, several entry inhibitors specifically blocking virus-cell attachment and fusion events have been evaluated preclinically as potential microbicides, including CCR5 antagonists (e.g., maraviroc or PSC-RANTES), lectins (e.g., Cyanovirin-N or Griffithsin, both of which bind to mannose moieties on HIV-1 gp120), and monoclonal antibodies (mAbs) that inhibit HIV-1 binding or entry through binding to viral envelope glycoprotein gp120, gp41, cellular receptor CD4 or coreceptor CCR5 [2], [3], [5].

Sifuvirtide (SFT) is a new HIV-1 fusion inhibitor whose design was based on the three-dimensional structure of the HIV-1 gp41 fusogenic core conformation [6]–[8]. In comparison with enfuvirtide (T20), the only approved HIV-1 fusion inhibitor by the U.S. Food and Drug Administration [9], SFT is more potent against infections by a wide range of primary and laboratory-adapted HIV-1 strains, including those resistant to T20 [8], [10]. SFT also exhibited good safety and tolerability in Phase Ia clinical study in which volunteers were dosed by subcutaneous injection [8]. Therefore, SFT represents a promising active component for microbicide development. Since SFT has not been tested for its anti-HIV-1 efficacy and biosafety in a gel formation which will be the case for the application in the genital tract or rectum as a microbicide candidate, therefore, we formulated SFT with 1.5% hydroxyethyl cellulose (HEC), a universal gel which has been used in several vaginal gel formulations, and assessed its biosafety, efficacy and stability; An universal placebo gel was also included in parallel for evaluation [11]. The results showed that SFT in the gel formulation exhibited equivalent anti-HIV-1 activities to SFT in phosphate-buffered saline (PBS) solution *in vitro*, and SFT gel displayed good biosafety and stability in both *in vitro* and *in vivo* evaluation models. Consequently, these findings suggest the potentials of SFT gel formulation for intravaginal or intrarectal application as a microbicide to prevent the sexual transmission of HIV-1.

**Figure 1:**
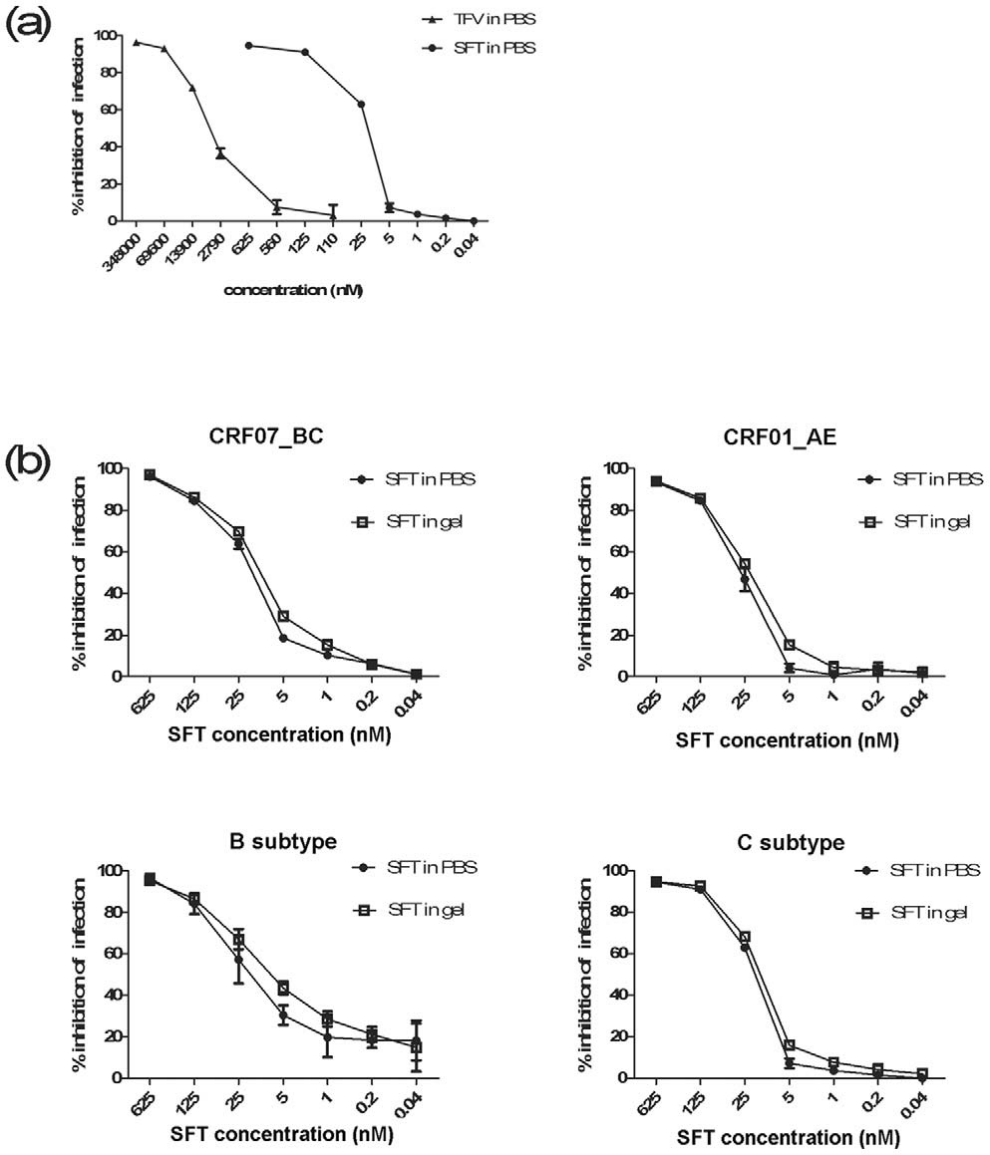
Efficacy of SFT gel against infections by HIV-1 pseudoviruses. (**a**) SFT is more effective *in vitro* against HIV-1 subtype C pseudovirus than TFV. The IC_50_ value of TFV in PBS against subtype C was 5.2 µM, whereas that of SFT in PBS was only 25.6 nM. (**b**) Efficacy of SFT in PBS or in 0.015% HEC gel to inhibit infection by HIV-1 pseudoviruses bearing the Env of CRF07_BC (upper left), CRF01_AE (upper right), B (lower left) and C (lower right), respectively. Data represent means ± SD from triplicate experiments. Student's *t*-test was performed to determine the significance of difference between various groups as shown in the graph, *p<0.05.

**Figure 2:**
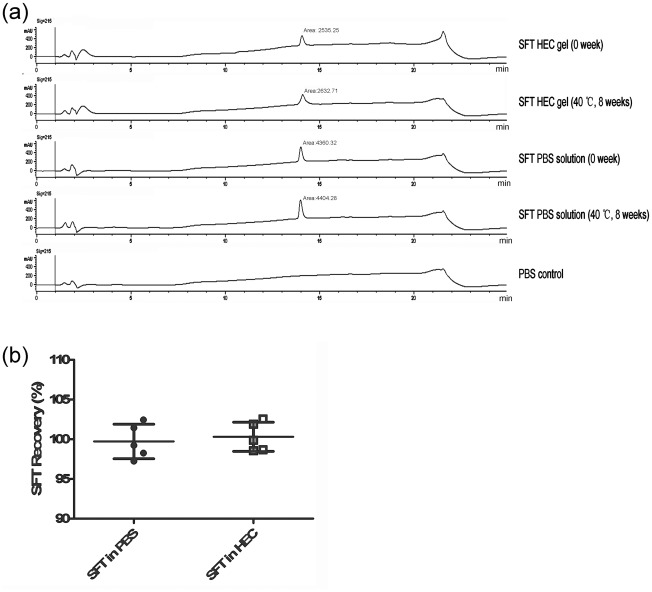
The content of SFT in HEC gel or PBS monitored by HPLC. (a) Representative chromatograms of SFT in 1.5% HEC and PBS. The retention time was 14 min for SFT. (b) Percent of SFT recovery from the 1.5% HEC and PBS after 8 weeks of storage at 40°C (n = 5, means ± SD).

**Table 1:** An accelerated stability study of SFT in the gel formulation.

Intervals	% Inhibition of HIV-1 infection^a^							
	SFT in gel				SFT in PBS			
	4°C	RT^b^	30°C	40°C	4°C	RT	30°C	40°C
Week 0	91.34±0.84	89.71±2.03	91.63±0.38	94.16±1.89	89.09±1.97	88.21±2.71	89.15±2.76	88.84±2.11
Week 1	80.54±1.04	96.84±1.15	92.09±1.70	94.91±2.88	94.69±1.64	92.08±1.23	92.62±2.31	94.48±1.41
Week 2	87.05±0.87	93.95±3.45	94.26±2.63	88.23±3.27	85.21±0.94	89.56±3.86	92.20±2.57	86.36±0.98
Week 3	89.76±0.16	87.28±1.88	89.51±0.49	88.77±2.35	88.71±3.41	84.64±1.47	90.68±0.11	81.73±4.66
Week 4	93.33±0.95	96.25±2.04	99.10±0.35	99.27±0.26	84.77±0.87	81.82±3.37	98.82±0.95	87.92±3.74
Week 5	93.80±1.63	95.03±1.69	93.92±1.10	87.92±2.72	92.61±2.88	93.79±2.43	90.54±0.68	92.67±1.24
Week 6	90.99±0.44	93.47±1.73	92.81±1.65	87.88±0.57	90.61±1.36	88.01±3.06	90.20±2.16	88.89±4.43
Week 7	93.22±4.43	90.64±1.70	89.66±4.01	92.32±1.02	93.16±1.44	92.06±1.01	93.67±0.82	92.80±1.80
Week 8	93.02±0.77	91.31±3.30	90.88±4.58	82.70±2.36	91.30±1.54	90.89±1.67	87.76±4.09	91.33±2.25

a Data represent mean ± SD (n = 3).

b RT: room temperature.

## Materials and Methods

### Reagents

Nonoxynol-9 (N-9) (catalog# SLN1945) and cellulose sulfate (CS) (catalog# SLC1798) were purchased from ScienceLab.com, Inc. (Houston, Texas); κ-Carrageenan (catalog# C1263) was purchased from Sigma-Aldrich (St. Louis, MO) and TFV (catalog# 14021945) was purchased from Molekula Limited (Gillingham, Dorset, UK). Sifuvirtide (SFT) was provided by FusoGen Pharmaceuticals, Inc. (Tianjin, China).

**Table 2:** Viscosity and pH profiles of SFT HEC gel stored at 40°C for 8 weeks.

	Samples		
Test	HEC gel^a^	SFT HEC gel (0 week)^b^	SFT HEC gel (at 40°C for 8 week)^b^
Apearance	Clear gel	Clear gel	Clear gel
pH	4.41±0.020	4.58±0.025	4.63±0.056
Viscosity^c^	5,800±21.5	5,830±18.9	5,810±15.5

a 1.5% HEC;

b 0.3 mM SFT in 1.5% HEC;

c Unit: mPa.s; Samples were tested in triplicate and the data were represented in mean ± SD.

For *in vitro* experiments on cell lines, SFT was dissolved in sterile PBS (pH = 7.4) at indicated concentrations, to which hydroxyethyl cellulose (HEC)(catalog# 434973, Sigma-Aldrich, St. Louis, MO) was added (final concentration of HEC: 0.015%), followed by continuously stirring for 45 min to allow all solutes to dissolve. For *in vivo* application in mouse vagina, SFT, TFV, and N-9 were first dissolved in PBS (pH = 4.5) at indicated concentration, to which HEC was slowly added to reach the final HEC concentration of 1.5% while the solution was rapidly stirred until a translucent gel was formed [12]. We used 0.015% and 1.5% HEC gel for *in vitro* and *in vivo* tests, respectively, based on an empirical rule [13], [14] that the concentration of the microbicide gel formation used in cell culture *in vitro* should be diluted 100-fold from the concentration used for *in vivo* test to facilitate the even spread of the product over the cell culture and to avoid interference with the cell growth in culture. . CS and κ-Carrageenan were dissolved at indicated concentration in pH 4.5 PBS without HEC to form gel formulations automatically. All gels were allowed to equilibrate overnight at room temperature and were retested for pH before intravaginal application as described below.

**Figure 3:**
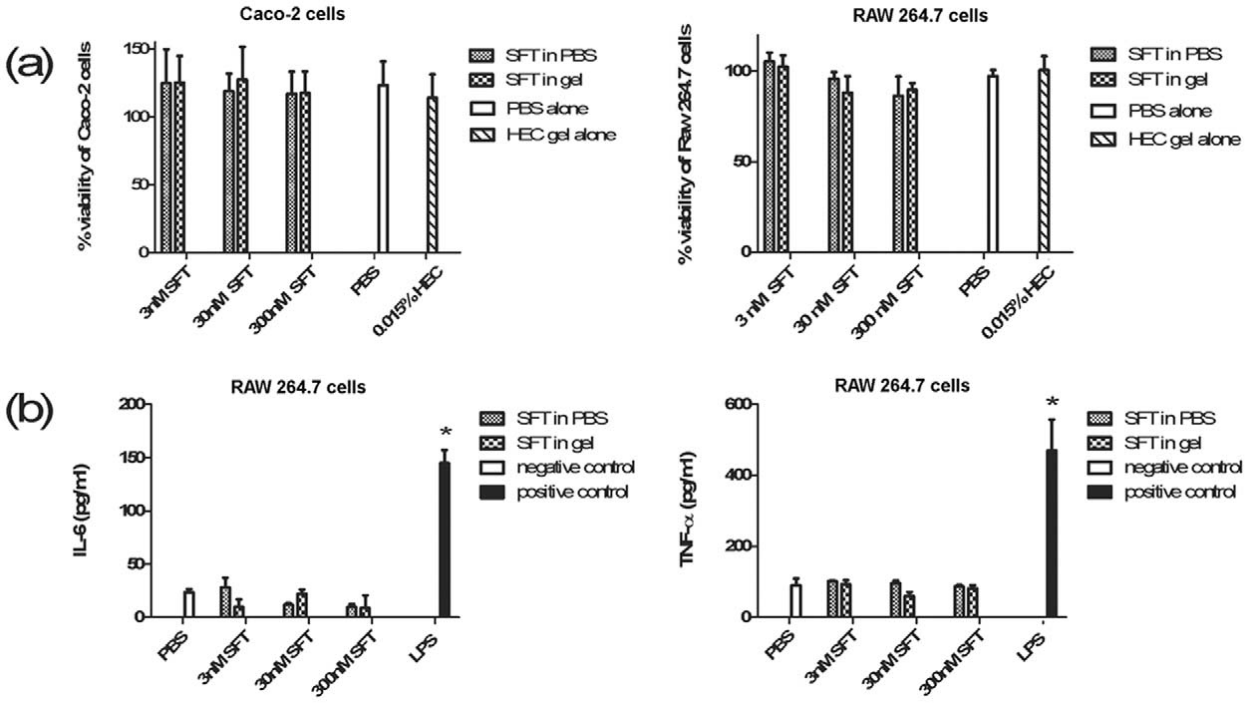
In vitro evaluation on the safety of SFT gel. (**a**) MTT assay was used to determine the cell viability of epithelial line Caco-2 (left) or macrophage cell line RAW264.7 (right) after the treatment of different concentrations (3 nM, 30 nM and 300 nM) of SFT in PBS solution or corresponding gel versions (formulated with 0.015% HEC). (**b**) Production of IL-6 (left) and TNF-α (right) in macrophage cell line RAW264.7 was analyzed by CBA assay after incubation for 12 h with the above-mentioned three concentrations of SFT in PBS solutions or 0.015% HEC gel. Data represent means ±SD from triplicate experiments. Student's *t*-test was performed to determine the significance of difference between the PBS-treated group and each microbicide candidate or LPS-treated groups, *p<0.05.

**Figure 4:**
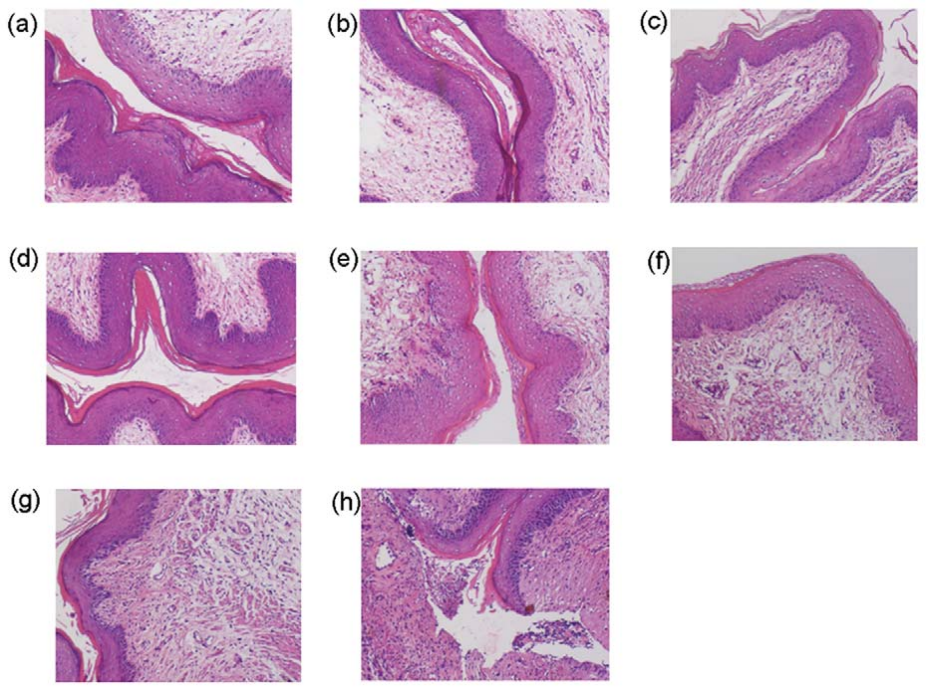
Histopathological examination of vaginal tissues after intravaginal application of SFT gel and other microbicide candidates. Slides were prepared from vaginal tissues treated with placebo (1.5% HEC gel) (**a**), 0.03 mM SFT gel (**b**), 0.3 mM SFT gel (**c**), 3 mM SFT gel (**d**), 1% TFV gel (**e**), 3% carrageenan gel (**f**), 6% CS gel-treated group (**g**), or 1% N-9 gel-treated group (**h**). Hematoxylin-eosin staining was used for all slides. Magnification, ×100.

**Figure 5:**
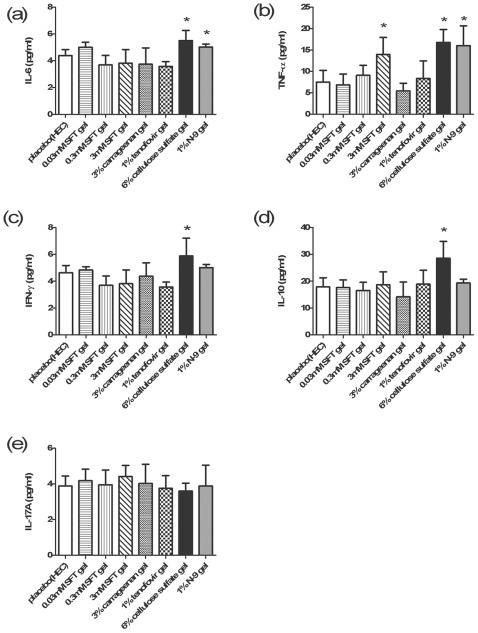
Inflammatory cytokines in CVLs after treatment of SFT gel and other microbicide candidates. Inflammatory cytokines, including IL-6 (**a**), TNF-α (**b**), IFN-γ (**c**), IL-10 (**d**) and IL-17A (**e**), were quantified in CVLs collected at 12 h post final dosage in the context of 3 consecutive days of intravaginal application of placebo (1.5% HEC), 0.03 mM SFT gel, 0.3 mM SFT gel, 3 mM SFT gel, 3% carrageenan gel, 1% TFV gel, 6% CS gel and 1% N-9 gel, respectively. The X-axis indicated the administration of different microbicide candidates; the Y-axis indicated the production of corresponding inflammatory cytokines. Data represent means ± SD from 5 mice per group. Student's *t*-test was performed to determine the significance of difference between the placebo gel control group and the treatment group for each microbicide candidate, *p<0.05.

### Cell Cultures

A human colorectal epithelial cell line, Caco-2, was kindly provided by ATCC (USA) and was cultured in Dulbecco's Modified Eagle Medium (DMEM); TZM-bl cells and mouse leukemic monocyte macrophage cell line RAW 264.7 were purchased from the Cell Bank of Type Culture Collection of Chinese Academy of Sciences and cultured in DMEM and RPMI 1640, respectively. All cell cultures were supplemented with 10% fetal bovine serum (FBS), 2 mmol/L L-glutamine, 100 U/mL penicillin, and 100 µg/mL streptomycin at 37°C/5% CO_2_.

### HIV-1 pseudovirus infection inhibition assay

In the present study, we tested antiviral activity of SFT in PBS or gel against viruses pseudotyped with Env of HIV-1 strains that are predominantly circulating in China, HIV-1 subtype B and subtype C. TFV, an NRTI that was proven in clinical trial to reduce the risk of women acquiring HIV-1 infection by 39% [4], was employed as a control. Briefly, four HIV-1 Env-pseudotyped viruses were produced in 293T cells by co-transfection with the expression plasmid encoding Envs of HIV-1 strains SVPB16 (subtype B), SVPC12 (subtype C), 32–72 (CRF07_BC), or SH188.6 (CRF01_AE), respectively, and HIV-1 backbone plasmid expressing the entire HIV-1 genome except Env, pNL4-3Δenv [15]. The pseudoviral particles were collected, titrated and stored at −80°C until use. For viral inhibition assay, approximately 1×10^4^ TZM-bl cells per well were plated into a 96-well plate in DMEM containing 10% fetal bovine and penicillin-streptomycin. After culture at 37°C for one day, pseudoviruses (100 TCID_50_ per well) were mixed with SFT or TFV in PBS solution or HEC gel formulation at the indicated concentration, and then the mixtures were added to the cells, followed by an incubation at 37°C with 5% CO_2_ for 48 h. The cells were then lysed in the presence of Bright-Glo (Promega), and relative luminescence was recorded by using a Victor 3 luminometer (PerkinElmer). The IC_50_ was then calculated.

### Accelerated stability study of SFT in microbicide gel

An accelerated stability study of SFT in microbicide gel was conducted as previously described [16]. Briefly, SFT at 0.3 mM was added into 1.5% HEC gel, and SFT-containing gel was stored at 4°C, room temperature, 30°C and 40°C, respectively, in 75% relative humidity (RH) for 1 to 8 weeks. Aliquots of samples were taken out every week and diluted by PBS to 80 nM SFT (an IC_90_ value against HIV-1 subtype C) for testing their inhibitory activity on HIV-1 pseudovirus subtype C infection, as described above. In addition, the stability of SFT in the gel was further analyzed by high-performance liquid chromatography (HPLC) on samples stored at 40°C for up to 8 weeks. Their viscosity and pH profiles were also determined as described below. The sample without incubation at 40°C (0 week) was used as a control.

### HPLC experiment

HPLC analysis of SFT peptide in gel were conducted on an Agilent 1100 LC system (Agilent Technologies) with a reverse-phase Ultra Aqueous C18 analytical column (2.1 mm×100 mm, 5 µm particle size) and a guard column (2.1 mm×12.5 mm, 5 µm particle size) which were purchased from Agilent Corporation. Samples were diluted in 10% acetonitrile (ACN), 0.1% formicacid (FA). The LC elution conditions were set as follows (all solvent percentages were volume fractions): mobile phase A, 0.1% FA in water; mobile phase B, 0.1% FA in ACN; time program, 0 min, 95% A/5% B; 1.0 min, 95% A/5% B; 10 min, 40% A/60% B; 12 min, 5% A/95% B; 15 min, 5% A/95% B; 16 min, 95% A/5% B; 25 min, 95% A/5% B. The flow rate was 200 µl/min at ambient temperature. Column oven temperature was 25°C. The detector was monitored with UV detection at 215 nm. Peak area (PA) was calculated by Chemstation software (Agilent Technologies) and recovery of SFT was calculated as “PA_sample of week8_/PA_sample of week0_×100”.

### Viscosity and pH determination of gel formulation

The viscosity of the gel formulations was measured by using a NDJ-5S Rotational Viscometer (Wuxi Engin International Corporation, Wuxi, China) with rotor #4 (60 rpm) at 25°C±0.1°C. The pH of the gels was determined at room temperature by using a 423 microprobe electrode (Mettler Toledo Instruments, Germany).

### Cytotoxicity tests in vitro

The effect of SFT gel or SFT PBS solution on the viability of Caco-2 cells or RAW264.7 cells was assessed by monitoring MTT metabolism using a colorimetric assay of cell survival which was carried out by the method of Denizot and Lang [17]. Briefly, cells were seeded at 5×10^4^ ml^−1^ in 96-well flat-bottom microtiter plates with different concentrations (3 nM, 30 nM and 300 nM) of SFT in 0.015% HEC gel or in PBS solution. After an incubation for 12 h at 37°C in a humidified 5% CO_2_, 50 µl of RAW264.7 cells in medium were sampled and stored at −80°C for further cytokine quantification assay as described below. After continuous incubation for 48 h, 10 µl of MTT (5 mg/ml) were added to the plate and incubated for 4 h at 37°C. Afterwards, the medium was removed, and the formazan, a product generated by the activity of dehydrogenases in cells, was dissolved in acidified isopropanol (0.4 N HCl). The amount of MTT formazan is directly proportional to the number of living cells and was determined by measuring the optical density (OD) at 490 nm by using a Bio Assay reader (BioRad, USA). The cell viability was calculated by the following equation: (OD treatment/OD control)×100. Experiments were carried out in triplicate.

### Safety evaluation in mouse model in vivo

All animal experiments were reviewed and approved by the Institutional Animal Care and Use Committee (IACUC) at Shanghai Public Health Clinical Center and were performed in accordance with relevant guidelines and regulations. Six- to eight-week-old pathogen-free outbred BALB/c female mice were purchased from Shanghai SLAC Laboratory Animal Co., Ltd. (Shanghai, China). All mice were hormonally synchronized with 2 mg of medroxyprogesterone acetate (Catalog# M1629, Sigma-Aldrich) 5 days prior to the treatment with candidate microbicides [18], [19]. For the *in vivo* safety study, mice randomized into eight groups (5 mice/group) were intravaginally treated with 40 µl of 1.5% HEC gel containing 0.03 mM SFT, 0.3 mM SFT, 3 mM SFT, 1% N-9, 1% TFV, 6% CS, 3% carrageenan, or PBS (vehicle control), respectively. The corresponding gel formulations were then delivered intravaginally every 12 h (twice daily) for three consecutive days. Twelve hours after the last treatment, cervicovaginal lavages (CVLs) were collected by washing the cervicovagina with 200 µl of sterile saline. The collected CVLs were treated with protease inhibitor (Complete Protease Inhibitor Cocktail; Catalog# 04693116001, Roche Applied Science, Indianapolis, IN) and centrifuged at 200×g for 10 minutes at 4°C. Supernatants were then collected and stored at −80°C for subsequent cytokine quantification assay. Finally, the mice were sacrificed, and the vaginal tissues were collected for preparation of mononuclear cells (MNCs) or for histological examination as described below.

### Histopathological examination

Formalin-fixed excised vaginal tissues were embedded within paraffin and transversely sectioned with a microtome. The slides were stained with hematoxylin-eosin and subjected to a blind evaluation for epithelial cell disruption and inflammatory responses.

### Cytokine quantification assay

Cytokines from RAW264.7 cell culture supernatants or cervicovaginal lavage fluid were determined by using a mouse cytokine CBA kit (Catalog# 560485, BD Biosciences, California) and analyzed on a FACSAria flow cytometer (BD Biosciences). Tumor necrosis factor (TNF)-α, interleukin (IL)-6, interferon (IFN)-γ, IL-17A and IL-10 were included in the cytokine panel. Standard curves were generated for each cytokine. Prior to examination, the boundary was set from 0–5000 pg/ml, and the lower detection limit for different analytes ranged from 0.03–16.80 pg/ml, as described in manufacturer's instructions.

### Statistical analysis

Data are presented as the mean ± standard deviation (SD). Statistical significance between different groups was calculated by the Student's *t*-test using GraphPad Prism, version 5.0 (San Diego, CA). Values of *p*<0.05 were considered statistically significant.

## Results

### SFT in gel formulation efficiently inhibit HIV infections in vitro

In previous studies, SFT showed strong inhibitory activity against a broad spectrum of HIV-1 strains, including those resistant to T20, with IC_50_ ranged from 2.68 to 47.78 nM [8]. In the present study, we showed that SFT was >200-fold more potent than TFV against HIV-1 subtype C pseudovirus infection (Figure 1a). The IC_50_ values of SFT in PBS against CRF07_BC, CRF01_AE, subtype B and subtype C were 13.3, 30.9, 5.9 and 25.6 nM, respectively, while those for SFT in 0.015% HEC gel were 9.8, 22.2, 3.4 and 12.4 nM, respectively (Figure 1b). These data suggested that HEC might have slightly enhanced the anti-HIV-1 efficacy of SFT in the gel. We and others have tested the inhibitory activity of SFT against HIV-1 infection in peripheral blood mononuclear cells (PBMCs) before and the results were well comparable with those obtained from the experiments using from TZM-bl cells [6], [8], suggesting that SFT in HEC gel is expected to be similarly effective against infection by primary HIV-1 isolates in humans.

### SFT formulated in HEC gel is stable

We tested the stability of SFT in HEC gel using an accelerated stability assay. As shown in Table 1, the anti-HIV-1 efficacy of SFT gels stored at the indicated temperatures was retained for up to 8 weeks. We then used HPLC to monitor the content of intact SFT in HEC gel and in PBS that were stored at 40°C for 8 weeks. As shown in Figure 2, SFT HEC gel and SFT PBS solution, both of which had been stored at 40°C for 8 weeks, exhibited the same peak shape and retention time, compared with those freshly prepared gels (0 week)(Figure 2a). The percent recovery of SFT at the end of the eighth week of storage at 40°C was 99.72%±2.18% in the PBS solution and 100.31%±1.84% in the HEC gel (Figure 2b), suggesting that there is no significant drug loss or generation of degradants after a sufficient period of time storage at a relatively high temperature.

Additionally, several physical and chemical properties of the SFT gel such as viscosity and pH in different conditions were also monitored. As shown in Table 2, the addition of SFT did not significantly change the viscosity of HEC gel. Furthermore, the viscosity and pH value of SFT HEC gel formulation that was stored at 40°C for was not changed remarkably. These results indicate that SFT is stable in microbicide gel formation for a sufficient period of time.

### Biosafety profile of SFT in HEC gel formulation

We first performed *in vitro* safety evaluation by using a cell line model. As shown in Figure 3a, both macrophage cell line RAW264.7 and epithelial line Caco-2 retained ∼100% viability with treatment of SFT gel or SFT PBS solution after 48 h of incubation, suggesting that neither SFT gel nor SFT PBS solution causes significant cell death. To determine the potential proinflammatory effect of SFT gel, we used SFT gels or PBS solutions to stimulate RAW264.7 cells for 12 h, respectively, and two proinflammatory cytokines include TNF-α and IL-6 could be detected in the cell culture supernatants. Compared to PBS control, neither SFT gel nor SFT PBS solution induced additional production of TNF-α or IL-6, whereas LPS (the positive control) significantly elicited the secretion of TNF-α and IL-6. These results suggest that both SFT PBS solution and SFT gel formulation have no proinflammatory effect on the cultured macrophage cells (Figure 3b).

To further investigate the biosafety of SFT gel on topical mucosa, a mouse model was used for *in vivo* evaluation. We first determined the impact of SFT gel on the integrity of mucosal tissues. Histopathological examination displayed that HEC gel alone-, TFV gel- and carrageenan gel-treated mice showed no damage or inflammatory response in the cervicovaginal tissues (Figure 4a, e, f), similarly, no obvious epithelial damage or inflammatory response was observed in the cervicovaginal tissues in all SFT gel-treated groups (Figure 4b, c, d). In contrast, N-9 gel treatment resulted in the disruption of epithelial cells and severe inflammatory response (Figure 4h). CS gel-treated mouse tissues displayed slight inflammatory response (Figure 4g).

We next determined the secretions of proinflammatory cytokines triggered on topical mucosal sites by those microbicide candidates mentioned above. While 1% TFV gel and 3% carrageenan gel, which have been clinically proven as safe microbicide formulations [4], [11], [20], did not induce any extra secretion of the five cytokines comparing to HEC gel alone; Similarly, 0.3 mM and 0.03 mM SFT gel also did not induce any enhancement of the cytokine production compared to HEC gel; 3 mM SFT gel upregulated secretion of TNF-α but not other cytokines. In contrast, 1% N-9 gel significantly upregulated the production of TNF-α and IL-6, 6% CS gel increased the secretion of TNF-α, IL-6, IL-10 and IFN-γ (Figure 5). Taken together, SFT gel has a potential to be developed as a safe and effective anti-HIV microbicide for preventing sexual transmission of HIV.

## Discussion

HIV-1 fusion/entry inhibitors, which target the initial step of HIV-1 life cycle, are promising microbicide candidates. As known, the mucus and the integrity of mucosal epithelial surface represent two effective barriers for invasive pathogens and prevent the occurrence of infections. It is believed that the disruption of the integrity of mucosal epithelial surface and the mucosal topical inflammation which could result in the enrichment of HIV-1 target cells and the dilution of vaginal mucus by ejected semen or increased volume of vaginal fluid during the intercourse might reduce the defense capabilities, thus facilitating HIV-1 spread across the mucosal membrane to establish infection [21], [22]. Therefore, the removal or disruption of those two barriers (the undiluted mucus and the intact mucosa) may be required for HIV-1 infection. It is rationalized that to build up the third barrier by employing the HIV-1 entry inhibitor will be also highly effective to block the invasion of HIV-1. Indeed, several entry inhibitors have been employed in the microbicide development, including CCR5 antagonists (e.g., maraviroc or PSC-RANTES), lectins (e.g., Cyanovirin-N or Griffithsin, both of which bind to mannose moieties on HIV-1 gp120), and several monoclonal antibodies that could interrupt the binding between virus and target cells. Under the consideration of building up the third barrier, large molecular entry inhibitors may be preferentially selected because they may be absorbed slowly and could extend their half-life at the mucosal sites. Another advantage of the slowly absorbed molecule over the rapidly absorbed drug as a microbicide is its slow absorption of the drug into blood, thus causing less systemic toxic effect. Its disadvantage is that under certain conditions, it may reach the target cells beneath the epithelium after the virus.

SFT is a more potent large fusion/entry inhibitor against HIV-1 strains, including those resistant to T20, when compared to T20 [8], and is attractive to be used as active component in microbicide candidate. In the present study, we investigated the anti-HIV-1 efficacy of SFT in a gel as formulated in microbicide, and showed that SFT in HEC gel retained its potent anti-HIV-1 activities, not only against subtype B or C, but also against CRF07_BC and CRF01_AE which are predominantly circulating in China, suggesting that it is likely that SFT would be effective when used in populations in China and other regions as a gel formulation. Since it is important for SFT to stabilize itself *in vivo*, we test the stability of SFT in gel at or above body temperature, our data showed that the SFT in gel could be stabilized for at least eight weeks at the temperature as high as 40°C, indicating that this product has the potential to stay at the mucosal site for a prolonged period. However, the continuous exchange between body fluid and mucosal surface and the possible appearance of enzyme at the mucosal microenvironment may greatly reduce the half-life of this product at the mucosal site. Our stability data are also applicable for the storage and transportation of this product, and indicated that this product will be much less dependent on cold-chain, thereby reducing the cold-chain related costs.

The safety profile of a microbicide candidate should be carefully evaluated before moving the candidate into clinical trial. As we mentioned above, the removal of two natural barriers at mucosa will increase the risk to acquire HIV-1 infection, therefore, anti-HIV-1 microbicides should not cause the disruption of mucosa integrity or inflammatory responses. Previous studies demonstrated that the systemic application of SFT was well tolerated without any severe adverse events in Phase Ia clinical trial [8]. In the present work, we evaluated the safety of SFT gel on topical mucosal site by using both cell line and mouse models. Data showed that SFT gel displayed good safety profiles in the *in vitro* assay and caused no histopathological alterations to vaginal epithelium in the mouse model. However, 3 mM SFT gel could upregulate the secretion of TNF-α in CVLs, suggesting that SFT concentrations over 3 mM should be excluded in further preclinical (e.g., evaluation by rhesus model) or clinical evaluations. Intriguingly, TNF-α was observed to be the cytokine most commonly and significantly upregulated, irrespective of treatment compound (N-9, CS or 3 mM SFT), suggesting that TNF-α is a sensitive and general inflammation marker to evaluate mucosal toxicity induced by different microbicide candidates.

Overall, the present study provides evidences that the SFT gel is a promising vaginal microbicide candidate and could be employed to build up the third barrier at the mucosal site. Though it is speculated that SFT may possess a prolonged half-life *in vivo*, a real-time monitoring will be required to determine its pharmacokinetics. In addition, SFT as an entry inhibitor is likely to exert its synergistic effect with other classes of antiretroviral drugs, such as reverse transcriptase inhibitor and protease inhibitor, and thereby be employed in the development of more potent microbicide candidate.
